# Antimicrobial Nanomaterials Based on Halloysite Clay Mineral: Research Advances and Outlook

**DOI:** 10.3390/antibiotics11121761

**Published:** 2022-12-06

**Authors:** Marina Massaro, Rebecca Ciani, Giuseppe Cinà, Carmelo Giuseppe Colletti, Federica Leone, Serena Riela

**Affiliations:** Dipartimento di Scienze e Tecnologie Biologiche, Chimiche e Farmaceutiche (STEBICEF), V.le delle Scienze, Ed. 17, 90128 Palermo, Italy

**Keywords:** clay minerals, halloysite nanotubes, antibacterial, wound healing, orthopedic implants, food packaging, pest control

## Abstract

Bacterial infections represent one of the major causes of mortality worldwide. Therefore, over the years, several nanomaterials with antibacterial properties have been developed. In this context, clay minerals, because of their intrinsic properties, have been efficiently used as antimicrobial agents since ancient times. Halloysite nanotubes are one of the emerging nanomaterials that have found application as antimicrobial agents in several fields. In this review, we summarize some examples of the use of pristine and modified halloysite nanotubes as antimicrobial agents, scaffolds for wound healing and orthopedic implants, fillers for active food packaging, and carriers for pesticides in food pest control.

## 1. Introduction

Infectious diseases, caused by microorganisms that are transferred directly or indirectly from one person and/or animal to another, are still a major healthcare problem [1]. They are indeed one of the major causes of death worldwide, particularly in seniors and young children in middle and low-income countries. In the last few years, several efforts have been devoted to the identification and control of most infectious diseases as well as the improvement of technologies to fight infections (bacteria, viruses, parasites, fungi, and so on).

In modern medicine, antibiotics are the most commonly used antimicrobial agents for the treatment of infections caused by pathogens. Unfortunately, pathogenic microbes, which mutate continuously, can develop mechanisms to evade antibiotics, increasing the cause of infection and decreasing the usefulness of their treatment. Consequently, antibiotic resistance is increasing, and it is a top global public health threat [2,3].

Nanotechnology offers a great opportunity to develop innovative materials that, due to their physicochemical properties, can efficiently be used as antimicrobial agents for several applications [3,4].

In this context, clay minerals, a class of nanomaterials, are considered a “godsend” for human beings because they are easily available, possess very low cost, are environmentally friendly, biocompatible, and capable of crossing cellular membranes [5].

**Figure 1:**
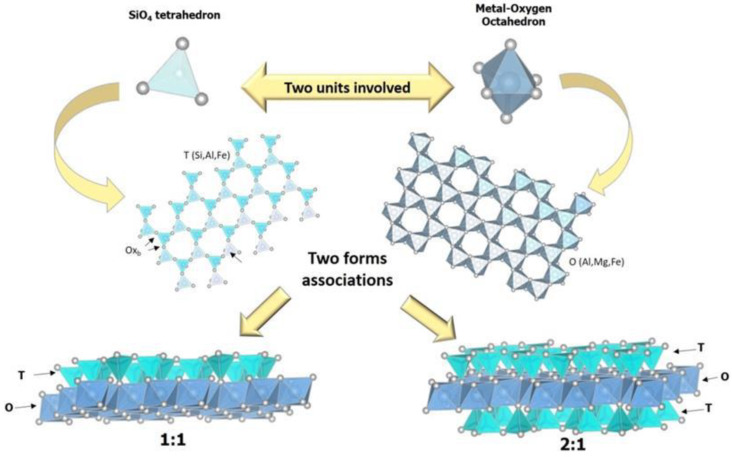
Representation of 1:1 (TO) and 2:1 (TOT) arrangements in clay mineral structure. Reproduced with permission from [8].

Clay minerals, derived from the chemical weathering of other silicate minerals [6], are natural layer-type phyllosilicates [7]. The different arrangements of tetrahedral (T) and octahedral (O) sheets allow clay minerals to be classified into different groups, the main ones being 1:1 and 2:1. In particular, in TO-type structure (1:1), tetrahedral silicon sheets are bonded to octahedral aluminum sheets belonging to this group of phyllosilicates, such as kaolinite and halloysite (the most representative clays of the kaolin group). On the contrary, the structure of the TOT type (2:1) is constituted by an octahedral sheet of Al^3+^, Fe^3+^, or Mg^2+^ sandwiched between two tetrahedral sheets of Si^4+^ or Al^3+^. Montmorillonite, hectorite, (expanding clay minerals representative of smectite group), and illite (a non-expanding clay mineral representative of the mica group) are examples of clay minerals belonging to this class (Figure 1).

**Figure 2:**
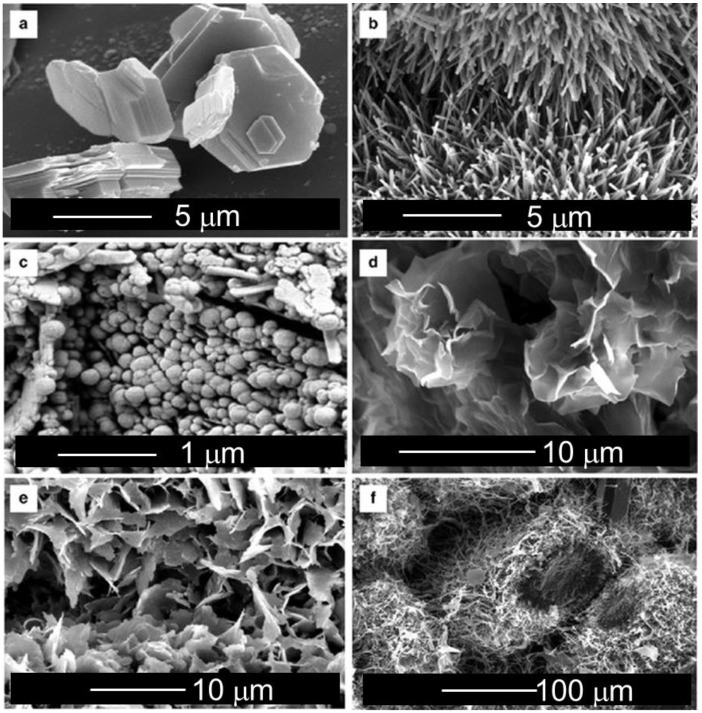
SEM images of clay minerals: (**a**) pseudohexagonal crystals of kaolinite; (**b**) tubular crystals of halloysite; (**c**) spheroidal crystals of halloysite; (**d**) wavy subhedral montmorillonite crystals [9]; (**e**) flaky illite crystals; and (**f**) fibrous illite. Reproduced with permission from [10].

Furthermore, clay minerals contain some water molecules that are confined both on the internal surface and in the clay interlayer. According to the TO-type structure and the intercalated water molecules, it is possible to observe different morphologies (Figure 2).

**Figure 3:**
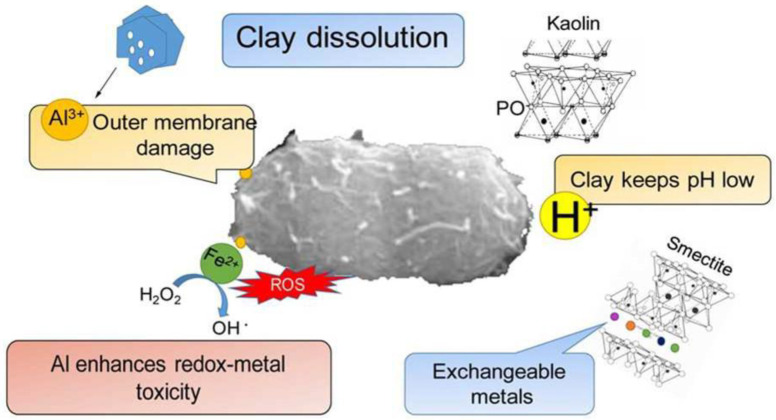
Cartoon representation of mechanisms that occur in the antibacterial activity of Al^3+^ and Fe^2+^ species of some clay minerals and bacteria. Reproduced with permission from [13].

Iron-rich clay minerals are traditionally classified as “healing clays”, mainly due to their capacity to release metal ions, such as Fe^2+^ ions, which are responsible for antimicrobial activity. Indeed, Fe^2+^ ions produce reactive oxygen species (ROS) that can damage membranes and DNA [11]. In addition, the presence of Al^3+^ ions increases the toxicity of the clays because Al can rearrange the membrane structure in ways that favor its oxidation [12,13] (Figure 3).

Recently, French green clay was reported to be used in the treatment of Buruli ulcer, a necrotizing fasciitis caused by *Mycobacterium ulcerans* [14]. Other clays, such as those belonging to the smectite group, can exert some bactericidal activity because of their cation exchange capability and swelling properties [14]. In this context, for example, several synthetic clay minerals were prepared by exchanging their native ions with known antibacterial ones such as Ag(I) ions by utilizing the cation exchange properties [15,16]. In this way, the Ag(I) ions were sustained release from the interlayer spaces ensuring long-term antibacterial effectiveness. 

**Figure 4:**
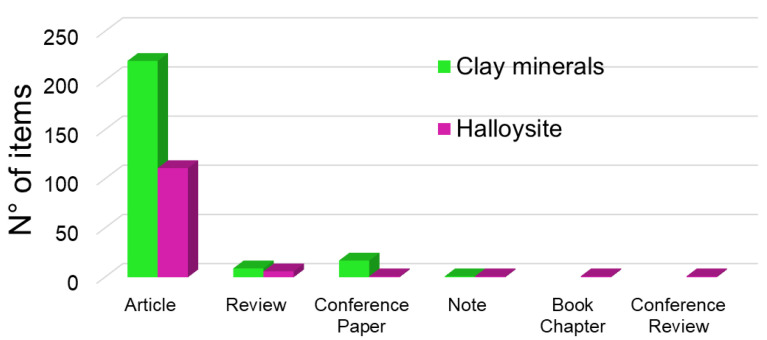
Number of publications (papers, reviews, conference papers, notes, conference reviews) obtained by searching Scopus (October 2022) for the terms antibacterial activity and clay minerals and halloysite, respectively.

In the last 20 years, halloysite has been widely used in biomedical applications due to its ability to act as a nanocontainer for biocides, reducing the amount of antibiotics used due to their controlled time-extended release. Due to these features, there has been more and more interest from the scientific community in the use of this kind of clay for antibacterial purposes, as testified by the increasing number of publications related to this topic (Figure 4).

In this review, we summarize the latest research about the functionalization of halloysite to develop nanomaterials that have found application for antibacterial protection, as antimicrobial agents for orthopedic and dental implants, as scaffolds for wound healing, in the active food packaging field, and as a carrier for pesticides in food pest control.

## 2. Halloysite Nanotubes Based Antimicrobial Materials

Halloysite is a natural phyllosilicate clay belonging to the kaolin group that shows an Al:Si ratio of 1:1 and a general formula of Al_2_Si_2_O_5_(OH)_4_∙nH_2_O. Typically, it is naturally found as nanotubes and therefore is usually referred to as halloysite nanotubes (HNTs). HNTs are constituted by 10–15 aluminosilicate bilayers, with a spacing of approximately 0.72 nm. The arrangement of the sheets generates an external surface composed by siloxane (Si–O–Si) groups and a lumen constituted by a gibbsite-like array of aluminol (Al–OH) groups. Furthermore, the rolling process causes some structural defects the also be present at the HNTs’ edges in the form of some Al–OH and Si–OH groups. The different chemical composition causes the tubes to undergo ionization in aqueous media in an opposite way, generating tubes with inner and outer surfaces oppositely charged across a wide pH range. In particular, the lumen is positively charged, whereas on the external surface there is a permanent negative charge. 

**Figure 5:**
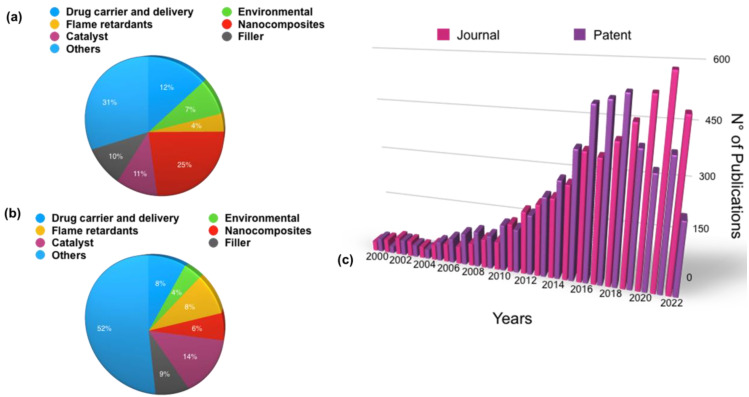
Comparison of the number of (**a**) scientific publications and (**b**) patents on the different applications fields of halloysite; (**c**) distribution (%) of scientific publications in “patent” and “journal” for halloysite. Data analysis of publications in October 2022 was performed using the SciFinder Scholar search system using searches for “Document type” the “Journal” and “Patent”, respectively.

By exploiting the different chemical composition and the different surface charges, HNTs can be modified, resulting in different nanomaterials with tunable properties that have found applications as fillers in polymeric matrices [17,18,19], drug carriers and delivery systems [20,21], supports for metal nanoparticles for catalytic purposes [22,23,24,25], and so on [26,27] (Figure 5a,b). The growing number of halloysite-related publications and patents attests to the clay’s growing popularity. It is noteworthy that the number of publications is comparable to that of patents, indicating an actual involvement of academia beyond industrial applications (Figure 5c). 

HNTs are biocompatible materials, and several in vitro and in vivo studies have assessed the non-toxic nature of this clay mineral. Halloysite, indeed, was found to be nontoxic for different cells [28,29], model organisms [30,31], and yeast cells [32]. Furthermore, it was found that by feeding HNTs to different animals, such as chickens and piglets, no toxic effects were observed [33,34].

Recently, an in vivo study was reported that allowed the authors to estimate the maximum concentration of HNTs that could be administered without observing toxicity. It was discovered that prolonged oral administration of 50 mg of HNTs per body weight for up to 30 days caused aluminum accumulation in mice lungs, resulting in pulmonary fibrosis [35]. 

**Figure 6:**
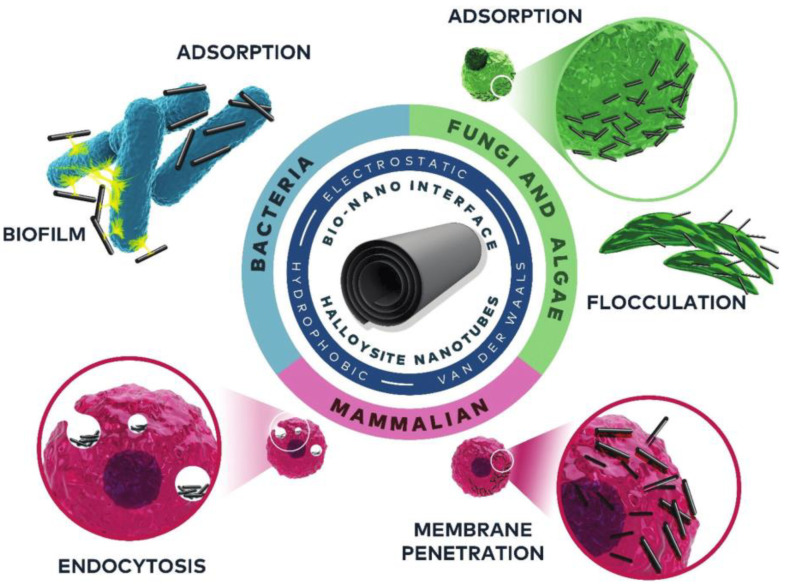
A scheme of the nano–bio interface of HNTs and cells along with the relevant mechanisms and interactions. Reproduced with permission from [36].

HNTs can interact with cells in different ways, some of them are driven by electrostatic (attraction) and/or hydrophobic interactions and/or van der Waals forces. On the contrary, the cells interact with HNTs depending on their nature. For example, while bacteria incorporate HNTs into their biofilm structure, in mammalian cells HNTs are uptaken through their membrane, whether via endocytosis or mechanisms where actin filaments are reported (Figure 6). 

Due to its intrinsic properties, halloysite, in contrast to some other clays, cannot be considered an antibacterial nanomaterial. It, indeed, lacks interlayer cation exchange properties and does not possess the ability to release metal ions, properties that are fundamental to exerting some bactericidal effects [36], as was already discussed. However, by suitable modification of the surfaces, it is possible to obtain nanomaterials with promising antibacterial activities. Furthermore, because HNTs possess an empty lumen, they have been used as nanocontainers for different antibiotics, obtaining nanomaterials that are used to treat common pathogens for different applications (Table 1) [37,38].

For example, the HNTs surfaces’ modification with three different charged surfactants, namely sodium dodecyl sulphate (SDS), acetyl trimethylammonium bromide (CTAB), and Tween 80, which are, respectively, anionic, cationic, and non-ionic surfactants, allowed the authors to synthesize nanomaterials that possess enhanced antibacterial activity depending on the surfactant used [60]. In particular, the toxicity of all nanomaterials was investigated against three phytopathogenic bacteria (*A. tumifeciens*, *X. oryzae*, and *R. solanacearum*) by calculation of the minimum inhibitory concentration (MIC), bacterial growth inhibition, cell membrane integrity loss, inhibition of biofilm formation, and reactive oxygen species production. Experimental findings showed that, among all surfactants, the modification of HNTs with CTAB produced the most efficient nanomaterial in suppressing the growth, inducing higher ROS production, disrupting the cell membrane integrity, and inhibiting biofilm formation. 

Conversely, the functionalization of HNTs with SDS was useful to obtain dispersible nanomaterials that, after the immobilization of AgNPs, were used as filler for carrageenan films [61]. The antimicrobial activity of the obtained nanocomposite was evaluated against two foodborne pathogen bacteria, *L. monocytogenes* and *E. coli,* by a total viable cell count method. This experiment showed that the modification of HNTs with SDS was a valuable strategy for obtaining a carrier of AgNPs, which confer good antibacterial activity to the polymer where it is dispersed.

Phosphomolybdic acid (PMo) was loaded into HNTs to synthesize nanomaterials with bactericidal properties, without the use of antibiotics [62]. The actual confinement of the PMo inside the tubes was verified by TEM analysis coupled with an EDX probe, and release experiments showed that the total amount of acid loaded in the tubes was released after 135 min. The so obtained nanomaterial was further loaded with AgNPs, resulting in a construct that showed good antibacterial properties against *P. aeruginosa*, *S. aureus*, and *A. baumannii*. In detail, the growth inhibition of *P. aeruginosa* and *S. aureus* was observed at 0.25 g L^−1^, whereas in the case of *A. baumannii* it was observed at 0.125 g L^−1^, with the MICs being at 0.5 and 0.25 g L^−1^, respectively. 

Tannic acid (TA) functionalized HNTs were used as scaffold for the immobilization of AgNPs onto clay in order to create a nanomaterial that can counteract antimicrobial resistance [54]. In vitro antibacterial tests on *S. aureus* ATCC 25923, *S.* Typhimurium, and *E. coli* ATCC 25922 highlighted that the nanomaterial possessed an MIC (31.25 μg/mL) against the QC strains and *S.* Typhimurium ca. 4-fold and >20-fold lower than that of AgNPs supported on tannic acid or HNTs/TA nanomaterial, respectively. 

Halloysite nanotubes were incorporated into HKUST-1, a Cu-based metal–organic framework, and used as filler for polyacrylamide hydrogels as human motion detection and strain sensors with high sensitivity, which have the advantages of repeatability, fast responsiveness, and, most importantly, antibacterial properties [63]. The authors of this work demonstrated that the nanocomposite hydrogels obtained showed excellent antimicrobial properties against *S. aureus* and *E. coli* as a consequence of the release of Cu(II) ions from the hydrogel matrix, which, once absorbed by bacteria, acts as an oxidizing agent for organic components, destroying the cell wall and leading to microbial death. 

The modification with antibodies properly oriented onto covalently bound protein A proved to be a successful strategy for developing systems capable of recognizing target bacteria [64]. It was found that the modified HNTs possessed superior binding toward target bacteria (*E. coli*) in comparison to the non-modified control, as quantitatively assessed by high-throughput flow cytometry. 

### 2.1. Orthopedic Implants

The rising age and longevity of the population have led to the implementation of primary arthroplasties worldwide. A consequence of this treatment is often represented by the occurrence of some infections, depending upon the type of bacteria involved and whether the infection is acute or chronic [65].

Over the years, to avoid bacterial infections, different biomaterials have been designed and engineered to ensure antibacterial protection. In this context, halloysite is an emerging filler that can be successfully used.

Lvov et al. (2012) investigated the possibility of using HNTs as a filler for poly-(methyl methacrylate) (PMMA), which has long been used as bone cement. To avoid bacterial infections, the authors of this study loaded HNTs with gentamicin, obtaining, after inclusion in the polymeric matrix, a nanocomposite with good mechanical strength and sustained release of the active ingredient [66]. Antibacterial tests highlighted that the gentamicin release inhibited the bacterial growth of *E. coli* and *S. aureus*, which were chosen as models.

A 3D-printed poly-ε-caprolactone (PCL) filled with HNTs and hydroxyapatite (HA) nanocomposite was fabricated by Riool et al. to release gentamicin sulfate (GS) when used as a coating for weight-bearing materials [67]. Specifically, the nanocomposite was obtained by mixing PCL, HNTs, HA, and GS, and the obtained mixture was subjected to fused filament fabrication (FFF) 3D printing technology to obtain a nanomaterial in the shape of a bone fixation plate. The nanocomposite obtained was intended to be applied to replace a mouse femur. The implant obtained was tested in in vitro, ex vivo, and in vivo experiments to study its antimicrobial efficacy. The experimental results highlighted the potentiality of this scaffold, which in the future can serve to produce load-bearing implantable devices with specific drug release properties.

### 2.2. Dental Implants

Nowadays, it is estimated that about 3.5 billion people worldwide suffer from oral diseases [68]. Often, to address this, it is necessary to resort to some dental implants and replacement procedures that, similarly to the orthopedic ones, are affected by bacterial infections.

In this context, Bottino et al. developed an injectable chlorhexidine (CHX)-loaded HNTs-modified GelMA hydrogel for dental infection ablation. GelMA is a photocrosslinkable gelatin methacryloyl [69], a polymer often used in regenerative engineering because of its good cell−tissue affinity and degradability in the presence of matrix metalloproteinases. The good antibacterial activity of the nanocomposite was tested on different pathogens associated with secondary endodontic infection (Figure 5). In addition, an in vivo test on stem cells from human-exfoliated deciduous teeth and the study of an inflammatory response using a subcutaneous rat model revealed good cytocompatibility with the hydrogel.

**Figure 7:**
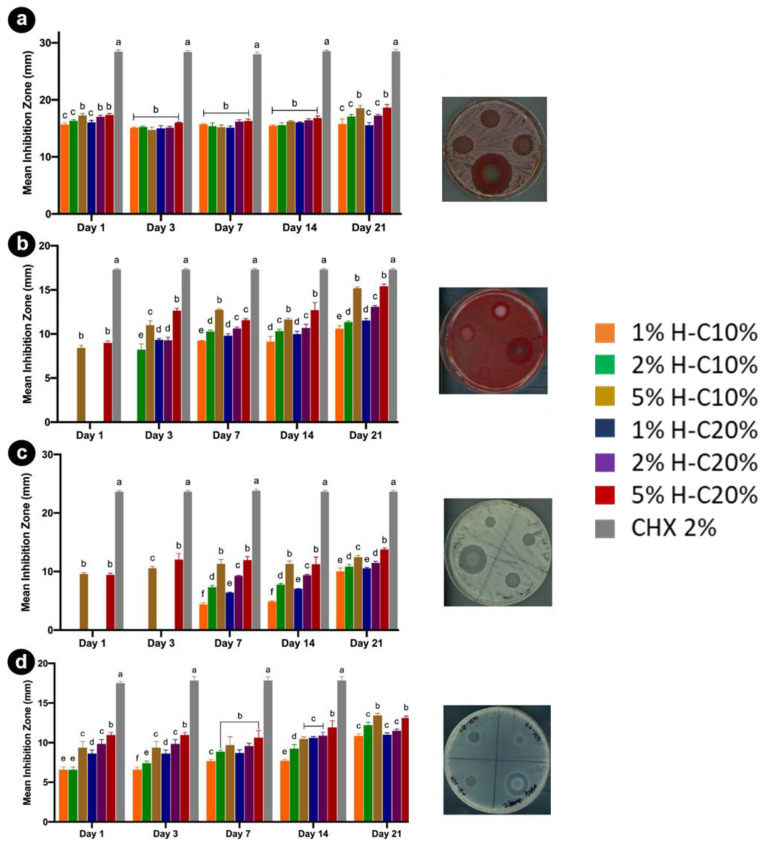
Results from the agar diffusion assays are represented as a mean inhibition zone (in mm) against the different pathogens tested. (**a**) *A. naeslundii*; (**b**) *F. nucleatum*; (**c**) *C. albicans*; and (**d**) *E. faecalis* over time. Distinct letters indicate statistically significant differences between the groups when compared with the control. Reproduced with permission from [69].

Similarly, chlorhexidine was loaded into HNTs, and the obtained nanomaterials were used as fillers for a dental resin [70]. The experimental findings show that the incorporation of different percentages of filler in the resin produced a nanocomposite with enhanced mechanical properties. In addition, it shows a slight decrease in curing depth and degree of conversion values, which are indicative of its durability. Biological assays showed no cytotoxicity on NIH-3T3 cell lines, and most importantly, antibacterial test on a strain of *Streptococcus mutans* highlighted the good antimicrobial activity of the nanocomposite (Figure 7).

### 2.3. Halloysite Based Nanomaterials for Wound Healing

Wound healing is a very complex process that occurs in subsequent or overlapped phases, involves a series of events, and requires the intervention of various mediators. Chronic skin wounds are lesions that fail to restore the skin’s anatomical and functional integrity, resulting in ulcers that take several years to heal. One of the most important issues that impairs the wound healing process is, of course, the occurrence of bacterial infections.

In this context, terpenoids structurally similar to carvacrol were loaded into HNTs, resulting in nanomaterials that performed well in cell-based scratch assays with a HaCaT cell monolayer on an in vitro artificial wound model for re-epithelialization and wound healing. In addition, the antimicrobial effects of the nanomaterials on the common pathogens that frequently colonize chronic wounds were also evaluated. The results of the experiments revealed promising antibacterial activity against four different Gram-positive and Gram-negative strains, namely *S. aureus* ATCC 43300, *S. aureus* ATCC 29213, *S. epidermidis* ATCC 35984, and *P. aeruginosa* ATCC 27853 [71].

Polymyxin B sulfate loaded on HNTs was used as filler for gelatin-based elastomers previously loaded with ciprofloxacin. As a result, a potentially useful biomaterial for wound dressing was obtained [18]. To validate the potentiality of the nanocomposite as antimicrobial agent, its antibacterial effects were studied on two different strains, *S. aureus* (Gram-positive) and *P. aeruginosa* (Gram-negative), commonly known to infect wounds, by evaluating the presence of inhibition zones around the bacterial discs. The experimental results showed good antimicrobial performance for at least 7 days, thanks to the slow release of both drugs from the nanocomposite.

**Figure 8:**
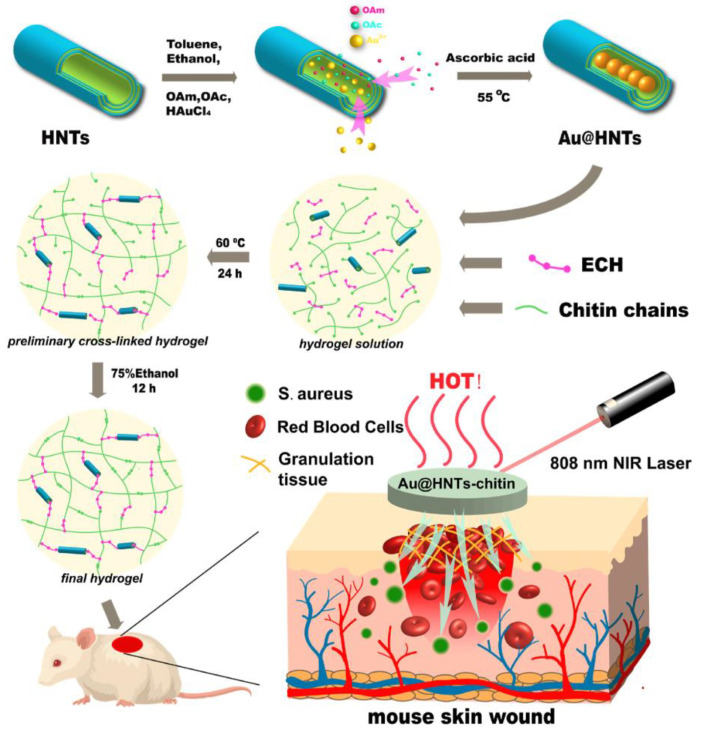
Schematic showing the preparation process and application of Au@HNTs-chitin hydrogel. Reproduced with permission from [72].

AuNPs encapsulated in HNT lumen were used to confer both photothermal and antimicrobial properties to chitin-based hydrogels for wound healing applications (Figure 8) [72]. The nanocomposite hydrogels possessed high cytocompatibility on mouse fibroblasts, and, by in vitro antibacterial experiments, it was demonstrated that, because of the photothermal properties, they showed high antibacterial ability towards *E. coli* and *S. aureus*. In addition, the chitin-based hydrogel showed the peculiarity of possessing high hemostatic performance in mouse liver and tail bleeding. In in vivo experiments, the authors of the study showed that wound infection healing results confirmed the healing-promoting effect of the hydrogel material.

### 2.4. Food Packaging

Nowadays, food spoilage due to microbial contamination represents a significant problem, which every year causes enormous economic loss. It is estimated that, in the United States alone, the wastefulness of food accounts for ca. 30–40% of the total food supply. Therefore, to prevent bacterial contamination, biofilms with antimicrobial properties should be prepared for active food packaging applications [73]. Among the different antimicrobial agents that have been used for this purpose, essential oils are the most-employed. Most of them have indeed been classified as “Generally Recognized as Safe” (GRAS) by the US Food and Drug Administration (FDA), and, in addition, they have been long been used as flavoring agents. However, they are highly flavoring, show high volatility, and show the tendency to be oxidized, and thus it is necessary to develop efficient carrier systems for their practical utilization.

In this context, thyme essential oil was loaded into HNTs, and then the nanomaterial obtained was mixed with flexographic ink and coated on paper for applications as food packaging materials [74].

Antimicrobial experiments on *E. coli*, for a 25-day treatment showed that the packaging paper filled with HNTs/TO nanomaterial, possessed a strong antimicrobial effect in the first 10 days. In particular, the packaging paper resulted in very high efficiency and was especially effective in eradicating *E. coli* within the initial 5 days, with the bacterial count reduced to ~1.5 log CFU cm^−2^.

**Figure 9:**
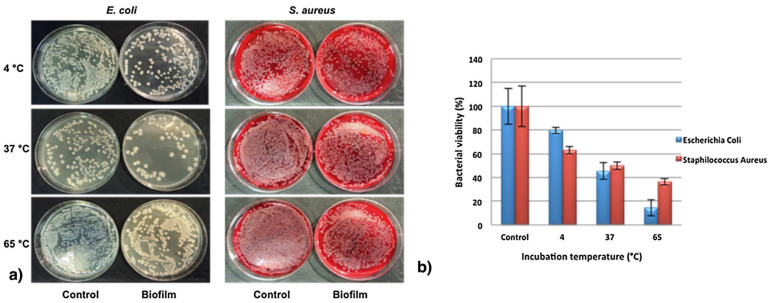
Antibacterial activity of pectin/HNT/PO films against *E. coli* and *S. aureus* at 4, 37, and 65 °C incubated for 30 min. (**a**) Photographs of petri dishes; (**b**) viability (expressed as% bacterial viability) of *E. coli* and *S. aureus* onto pectin/HNT/CB[6]/PO films after incubation at the three different temperatures. Reproduced with permission from [76].

Similarly, Gorrasi et al. [75] used rosemary essential oil loaded in HNTs as a filler for pectin matrix, while peppermint essential oil was loaded on cucurbit[6]uril (CB[6])-modified HNTs [76]. In this case, the HNTs/CB[6] nanomaterial was mixed by an optimized casting process into pectin, obtaining a nanocomposite with superior antioxidant and antibacterial activities. The in vitro antimicrobial activity of the nanocomposite was evaluated on *E. coli* and *S. aureus*, isolated from beef and cow milk, respectively (Figure 9), at three different temperatures. It was found that the percentage of bacterial viability for both bacterial strains was reduced at 65 °C compared to those at 37 °C and 4 °C. Of note, only 15% of the *E. coli* survived after the treatment at 65 °C.

**Figure 10:**
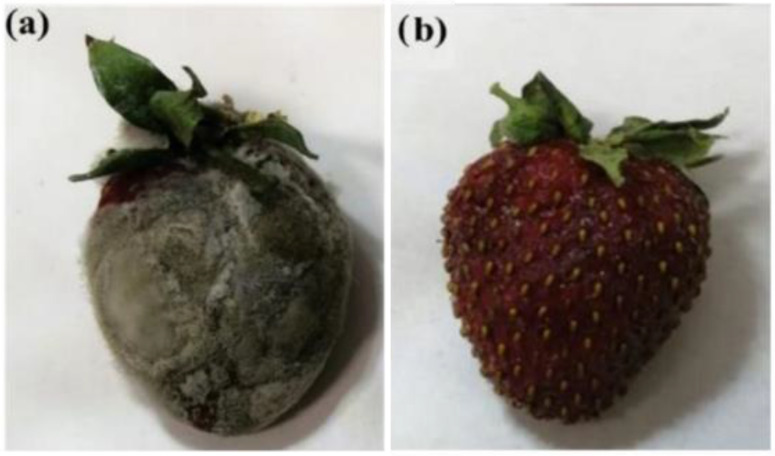
Fresh strawberry: (**a**) uncoated; (**b**) coated with pectin nanocomposite after 10 days of storage at room temperature, RH = 60%. Reproduced with permission from [77].

Following the same approach, grapefruit seed oil was encapsulated into HNTs’ lumen and then dispersed in a pectin matrix, obtaining a nanocomposite that was effective in the protection of fruits [77]. The authors, indeed, coated fresh strawberries with the film developed and stored them for 10 days at room temperature RH = 60%. The nanocomposite films prevented mold formation, extending the storage time of such fruit, in contrast to the uncoated strawberry, which showed mold after two days with a wrinkled and damaged appearance (Figure 10).

One of the most-used chemical species active in food packaging is represented by ZnO nanoparticles. It is indeed registered by the US Food and Drug Administration (FDA) (FDA, 2011) on the Generally Recognized as Safe (GRAS) list. Therefore, over the years, when the utilization of HNTs as filler for active food packaging applications is concerned, several efforts have been made to develop innovative nanomaterials/nanocomposites with ZnO.

In 2015, Pasbakhsh et al. reported the deposition of ZnO on HNTs to obtain a filler for poly(lactic acid) (PLA) films [78]. The nanocomposite obtained showed enhanced mechanical properties in comparison to the neat polymer, and most importantly, it possessed exceptional antimicrobial activities against *E. coli* and *S. aureus*.

Similarly, ZnO@HNTs nanomaterials with a nominal wt% ratio of ZnO to halloysite equal to 4 as filler for chitosan/polyvinyl alcohol (CS/PVOH) matrices were obtained [79]. The films were tested for their antimicrobial efficacy against four common food pathogenic bacteria, namely *E. coli*, *S. enterica*, *L. monocytogenes,* and *S. aureus*. To prove the biological activity, two different parameters were evaluated: the inhibitory activity, by measuring the diameter of the clear inhibition zone, and the bacterial growth inhibition. Both experiments showed enhanced antibacterial activity of the nanocomposite in comparison to chitosan.

Acid-treated HNTs were used as nanocontainers for cinnamaldehyde and as filler in alginate film [80]. The use of HNTs was helpful in slowing down the release of the active ingredient from the film. Kinetic release experiments, using isooctane as the release medium, to simulate fatty foods, showed that after 72 h, the filler containing HNTs still retained about 60 wt% of the total amount of cinnamaldehyde loaded. Antimicrobial tests highlighted the usefulness of HNTs in the nanocomposite; indeed, the hybrid nanocomposite demonstrated prolonged antimicrobial action on *E. coli* and *S. aureus* for at least four and five days more, respectively, in comparison to cinnamaldehyde simply dispersed in the alginate.

Similarly, using layer-by-layer (LbL) self-assembly technology [81], HNTs loaded with cinnamaldehyde were further functionalized with positively charged poly(allylamine hydrochloride) (PAH) and negatively charged poly(styrene sulfonate) (PSS). The goal of this kind of functionalization was to cloak the tubes with end-stoppers to avoid the fast release of the active ingredients. The authors of this study demonstrated that cinnamaldehyde was selectively released at a low pH value; therefore, the nanomaterial could be used to develop smart packaging for food protection. To prove this hypothesis, some antimicrobial tests on *S. aureus* and a pilot study of packed fresh wheat noodles with the developed nanomaterial were performed. It was demonstrated that the HNT-based nanomaterial showed good fumigant antimicrobial activity, and the total plate count, study of pH and color change, and environmental SEM characterization of the treated noodles highlighted that it can be effectively used to extend the shelf-life of fresh wheat noodles.

**Figure 11:**
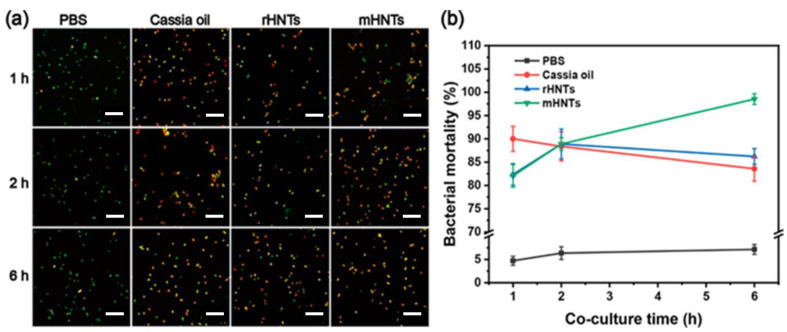
(**a**) Fluorescence images and (**b**) corresponding bacterial mortality data statistics of *S. aureus* co-cultured with different materials (PBS, cassia oil, rHNTs, and mHNTs). Scale bar 10 μm. Reproduced with permission from [82].

Recently, to solve the problems arising from the low water solubility and high volatility of some antimicrobial agents, an innovative strategy was adopted based on Pickering emulsion. In this context, properly modified HNTs were used to prepare emulsions based on cassia oil, selected as the oil phase [82]. To render the external surface of halloysite hydrophobic, and therefore to obtain stable emulsions, HNTs were firstly subjected to ball milling in a polytetrafluoroethylene (PTFE) jar. During the process, PTFE was transferred from the milling jar walls to HNTs surface, changing its hydrophilicity and electrical properties. Finally, the obtained nanomaterials were used as solid particles on the oil−water interface for preparing Pickering emulsions. Antibacterial experiments showed that the use of hydrophobic HNTs as an emulsifier of cassia oil enhanced its antibacterial properties towards *S. aureus* and *E. coli*. Bacterial growth kinetics experiments and live/dead bacterial viability assays (Figure 11) further confirmed the improved biological properties of the nanoemulsions and showed that cassia oil is slowly released from the HNTs. The results obtained in the present work open the doorway to the use of HNTs as emulsifiers for the preparation of Pickering emulsions for future applications in food protection.

### 2.5. Carrier for Pesticides

Population growth has necessitated increased food production, which has been hampered by climate change and agricultural crop pests, to name a few. Up until now, pest control has been a fundamental part of good manufacturing practice in food processing from an economic, hygienic, and regulatory viewpoint. Therefore, the development of systems capable of being carriers and gradually releasing the pesticides is crucial to reducing both their environmental impact and ensuring pest protection over time. In this context, halloysite, which has shown excellent eco-compatibility [83,84,85], represents an inexpensive carrier for several pesticides.

**Figure 12:**
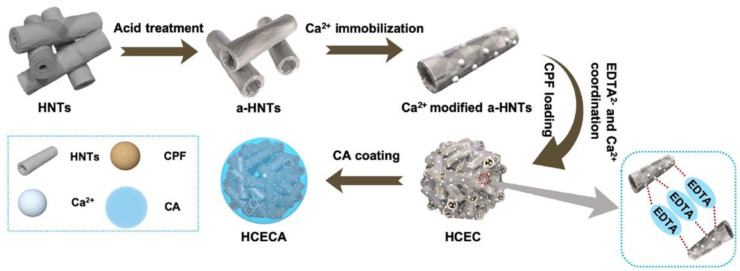
Schematic representation of the HNT/CPF pesticide. Reproduced with permission from [86].

Acid-treated HNTs were used as carrier for chlorpyrifos (CPF), a hydrophobic pesticide, followed by the coating of the tubes with alginate gels, used to slow down the release of the CPF from the tubes. To increase the loading of the active ingredient, a three-dimensional structure involving the acid-treated HNTs was also created via a step-by-step modification of the HNTs’ surface with Ca^2+^ and EDTA^2−^, exploiting their strong coordination interactions (Figure 12) [86].

In addition to the increase in loading efficiency and slow release of CPF, the synthesized nanopesticide, because of the strong interaction of alginate with plant leaves, shows a foliar adhesion property against rain rinsing that is strengthened by 86% in comparison to pristine CPF, thus reducing the overall environmental impact.

Similarly, CPF was loaded onto modified HNTs to develop a novel pesticide for the control of the growth of beet armyworm (which grows fastest at 35 °C) [87]. The modification of HNTs was achieved by the grafting of a thermo-responsive polymer, poly-isopropylacrylamide (PNIPAAM), followed by a polydopamine coating that was used to avoid fast release of CPF. The nanomaterial showed excellent thermosensitive release performance, with an average release rate of CPF at 35 °C ca. 2.5 times higher than that at 25 °C.

An emulsion of chlorantraniliprole (CAP) in xylene was added to an aqueous HNTs dispersion, forming a three-dimensional network structure that showed increased leaf adhesion, rain erosion resistance, and insecticidal effect in comparison to “free” CAP [88]. To test the insecticidal activity of the synthesized system, *S. frugiperda* was selected as a pest model. The experimental results showed an increased mortality in the presence of the HNTs based emulsion, indicating that the system is promising for future applications.

The loading of pyrethrum extract into HNTs’ lumen led to the synthesis of nanopesticides where, because of the presence of HNTs, the active ingredients are protected from UV light and slowly released over time. In addition, in vivo tests on two different insects, *G. mellonella* and *T. molitor*, chosen as pest models, showed that the nanomaterial was highly active on the first one at a half dose compared to a commercial pesticide [89].

## 3. Conclusions

The functionalization of halloysite nanotubes both by supramolecular approach and by covalent modification represents a valuable strategy for the development of low-cost and innovative antimicrobial agents for applications in several fields. The clay’s bio- and eco-compatibility, as well as its ease of availability and physicochemical properties, are important factors in the biomedical use of HNTs. Furthermore, the empty lumen of HNTs is useful for the loading of antimicrobial species, conferring on the clay the antibacterial properties required for future applications, such as topical wound treatment or active food packaging. At the same time, the encapsulation of active ingredients helps their sustained release over time and ensures their protection from photodegradation.

Scientific research on HNTs is still in progress, and every day innovative chemical strategies are proposed to expand the utilization of this clay.

Halloysite is a clay belonging to the kaolin group; conversely to “smectite” clays, it does not possess a high CEC capacity. Recently, to resolve this aspect, the covalent linkage of HNTs with another clay, specifically hectorite, to develop a nanomaterial that possesses both the typical characteristics of the two clay minerals, namely an empty lumen and expandible interlayer surfaces, which allow cation exchange, has been proposed [90].

The obtained nanomaterial was tested as a carrier agent for two antimicrobial species, ciprofloxacin and Ag(I) ions. Ciprofloxacin molecules were specifically loaded into the lumen of HNTs, whereas Ag(I) ions were found in the hectorite interlayers, as demonstrated by various techniques. The high loading of the two species obtained could be promising for the future application of the nanomaterial in infections where synergism between two antibacterial species is needed.

## Figures and Tables

**Table 1:** Different HNTs based antimicrobial nanomaterials and their relative applications.

Nanomaterial	Biocide	Pathogen	Application	Ref.
HNTs-NH_2_	Gentamicin	*E. coli*, *S. aureus* and *S. epidermidis*	Antibacterial	[39]
HNTs	Oregano essential oil	*E. coli*, *S. aureus*	Food packaging	[40]
HNTs	Carvacrol	*A. hydrophila*, *P. putida*, *L. monocytogenes* and *S. aureus*, *A. alternata*	Antibacterial	[41,42]
HNTs	CdS	*E. coli*, *S. aureus*	Antibacterial	[43]
HNTs/pectin	Salicylic acid	*Salmonella*, *P. aeruginosa*, *E. coli* and *S. aureus*	Food packaging	[44]
HNTs/pectin HNTs/alginate	Salicylic acid	*E. coli, S. typhimurium*, *P. aeruginosa* and *S. aureus*	Food packaging	[45]
HNTs/low-density polyethylene	Carvacrol and thymol	*E. coli*	Food packaging	[46]
HNTs/polyethylene	Carvacrol	*A. hydrophila*	Food packaging	[47]
HNTs-poly(4-vinylpyridine)	CuNPs	*E. coli*	Antibacterial	[48]
HNTs- poly(4-vinylpyridine)/polyethersulfone	AgNPs	*E. coli*, *S. aureus*	Antifouling and antibacterial	[49]
HNTs/chitosan	Norfloxacin	*E. coli*, *S. aureus*	Antibacterial	[50]
HNTs/chitosan/polyvinyl alcohol nanofibers	Benzocaine	*E. coli*, *S. aureus*	Antibacterial	[51]
HNTs/sodium alginate-poly (ethylene oxide) fibrous mats	Levofloxacin	*E. coli*, *S. aureus*	Wound dressing	[52]
HNTs/chitosan/pullulan	Rutin	*E. coli*, *L. monocytogenes*	Food packaging	[53]
HNTs/alginate	Cephalexin	*E. coli, P. aeruginosa* and *S. aureus*	Antibacterial protection	[54]
HNTs/polyethylene glycol	ClO_2_	/	Food packaging	[55]
HNTs/poly(lactic) acid	Clove essential oil	/	Food packaging	[56]
HNTs/chitosan	Clove essential oil	*B. mojavensis*, *E. coli*	Food packaging	[57]
HNTs/LDPE	Carvacrol	*E. coli*, *S. aureus*	Food packaging	[58]
HNTs/silk fibroin microfibers	Tetracycline hydrochloride	*E. coli*, *S. aureus*	Wound dressing	[59]
HNTs/poly(lactic) acid	Clove essential oil	/	Food packaging	[56]

## Data Availability

Not applicable.
